# A Small Number of HER2 Redirected CAR T Cells Significantly Improves Immune Response of Adoptively Transferred Mouse Lymphocytes against Human Breast Cancer Xenografts

**DOI:** 10.3390/ijms21031039

**Published:** 2020-02-04

**Authors:** Gábor Tóth, János Szöllősi, Hinrich Abken, György Vereb, Árpád Szöőr

**Affiliations:** 1 Faculty of Medicine, Department of Biophysics and Cell Biology, University of Debrecen, 4032 Debrecen, Hungary; tothgab@med.unideb.hu (G.T.); szollo@med.unideb.hu (J.S.); 2 MTA-DE Cell Biology and Signaling Research Group, Faculty of Medicine, University of Debrecen, 4032 Debrecen, Hungary; 3 Regensburg Center for Interventional Immunology, Dept. Genetic Immunotherapy, and University Hospital Regensburg, D-93053 Regensburg, Germany; Hinrich.Abken@klinik.uni-regensburg.de; 4 Faculty of Pharmacy, University of Debrecen, 4032 Debrecen, Hungary

**Keywords:** breast cancer, trastuzumab, chimeric antigen receptor, immunotherapy, cell therapy

## Abstract

HER2 positive JIMT-1 breast tumors are resistant to trastuzumab treatment in vitro and develop resistance to trastuzumab in vivo in SCID mice. We explored whether these resistant tumors could still be eliminated by T cells redirected by a second-generation chimeric antigen receptor (CAR) containing a CD28 costimulatory domain and targeting HER2 with a trastuzumab-derived scFv. In vitro, T cells engineered with this HER2 specific CAR recognized HER2 positive target cells as judged by cytokine production and cytolytic activity. In vivo, the administration of trastuzumab twice weekly had no effect on the growth of JIMT-1 xenografts in SCID mice. At the same time, a single dose of 2.5 million T cells from congenic mice exhibited a moderate xenoimmune response and even stable disease in some cases. In contrast, when the same dose contained 7% (175,000) CAR T cells, complete remission was achieved in 57 days. Even a reduced dose of 250,000 T cells, including only 17,500 CAR T cells, yielded complete remission, although it needed nearly twice the time. We conclude that even a small number of CAR T lymphocytes can evoke a robust anti-tumor response against an antibody resistant xenograft by focusing the activity of xenogenic T cells. This observation may have significance for optimizing the dose of CAR T cells in the therapy of solid tumors.

## 1. Introduction

Human epidermal growth factor receptor 2 (HER2) is overexpressed in 20–25% of breast cancer tumors [1]. HER2 expression is associated with an aggressive disease with a high recurrence rate and increased mortality [2]. Specific monoclonal antibody therapy has revolutionized the treatment of HER2 positive breast cancer since the FDA (U.S. Food and Drug Administration) approval of trastuzumab (Herceptin^®^) in 1998 [3]. The addition of trastuzumab to chemotherapy results in a lower rate of death after one year (22 percent vs. 33 percent, *P* = 0.008), longer survival (median survival, 25.1 vs. 20.3 months; *P* = 0.046), and a 20 percent reduction in the risk of death [4]. Despite the success, resistance to therapeutic antibodies is a clinical reality that affects the outcome of 60–80% of HER2+ breast cancer patients [5]. One of the underlying mechanisms is epitope masking by components of the tumor microenvironment (TME) such as the MUC4 (mucin 4) or the CD44/Hyaluronan complex [6,7,8,9,10,11].

The JIMT-1 cell line was established from the pleural metastasis of a breast cancer patient and has recapitulated the trastuzumab resistance of the original tumor in vitro and also in vivo if treatment of JIMT-1 xenografts SCID mice was initiated at a few hundred mm^3^ tumor volumes [12,13,14]. Our recent data indicate that simultaneous targeting of two epitopes on the HER2 molecule with clinical doses of trastuzumab and pertuzumab additionally improves the efficacy of antibody-dependent cellular cytotoxicity and thereby also the anti-tumor response; however, eventually all JIMT-1 xenografts become resistant to antibody treatment at a certain tumor size [15].

In such cases of antibody resistance, Chimeric Antigen Receptor (CAR) engineered T cells [16] represent an appealing option for improving the outcome for patients with advanced breast cancer [17,18,19,20]. Several tumor-associated membrane proteins are targeted in clinical trials by CAR T cells, including HER2 (NCT02547961, NCT02713984), CEA (NCT02349724) and mesothelin (NCT02792114). While no results have been disclosed of current HER2 targeting trials, the first reported clinical use of HER2 specific CAR T cells resulted in a serious adverse event following CAR T cell infusion [21]. In this trial, a HER2 positive colon cancer patient was treated with a large number (10^10^) of CD28-41BB costimulatory (3rd-generation) CAR T cells, which derived their antigen specificity from trastuzumab. The patient developed respiratory distress, followed by multiple cardiac arrests over the course of 5 days, leading to death. The death of this patient may have occurred due to the result of HER2 recognition of highly active and numerous anti-HER2 CAR T cells in the normal lung tissue that caused pulmonary toxicity and edema followed by a cytokine release storm causing multiorgan failure. The immune-mediated recognition of tumor antigens in normal tissues is referred to as “on-target, off-tumor” toxicity. It is thus clear from both preclinical experiments and clinical trials that while CAR T cell-based immune therapy has great potential to improve the outcome for patients with HER2 positive tumors, it still needs plentiful optimization.

Here, we report the generation of mouse T cells that are genetically modified to express a chimeric antigen receptor that consists of a HER2 specific single-chain variable fragment (scFv) derived from trastuzumab, a CD28 costimulatory endodomain, and a CD3z intracellular signaling domain. We demonstrate that these T cells recognize and kill HER2+ tumor cells in vitro and significantly improve the xenogenic immune response against human breast cancer even at very low numbers (17,500), resulting in complete tumor regression, and significant survival advantage. 

## 2. Results

### 2.1. Generation of Murine HER2 Specific CAR T Cells

**Figure 1:**
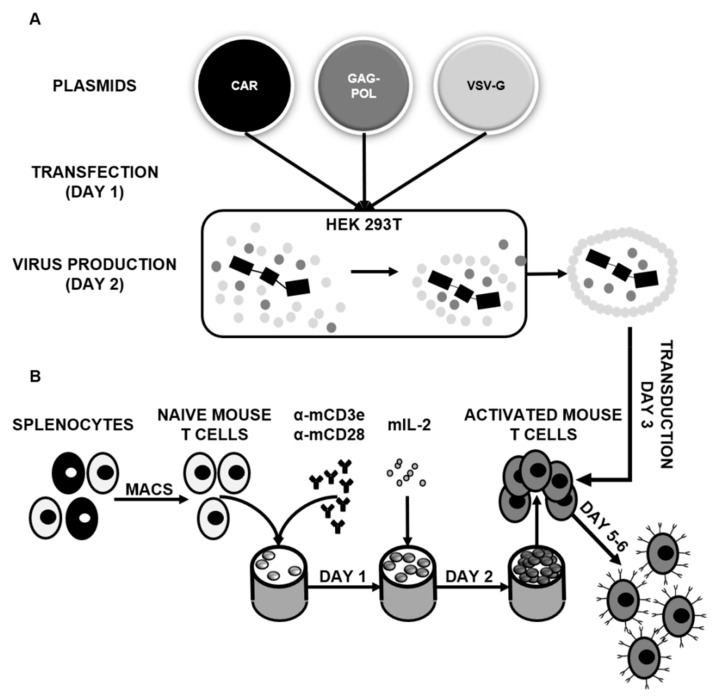
Genetic modification of mouse T cells with chimeric antigen receptors: (**A**) Scheme of retrovirus production. (**B**) Scheme of mouse T cell separation and activation.

To genetically modify mouse T cells (Figure 1), first, we generated VSVG-pseudotyped retroviral particles encoding HER2 specific chimeric antigen receptors (Figure 1A). T cells were isolated from the freshly dissected spleen of congenic Balb/c mice and activated by anti-mouse CD3e and anti-mouse CD28 antibodies. After 24 h, the medium was supplemented with mouse interleukin 2. Finally, activated mouse T cells were retrovirally transduced on RetroNectin-coated plates (Figure 1B).

**Figure 2:**
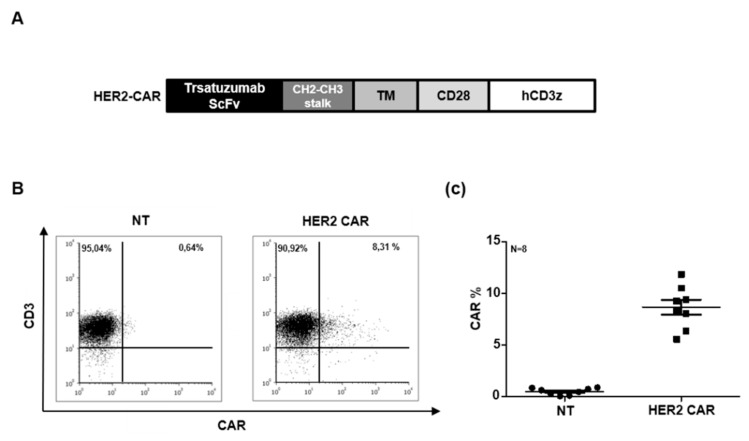
Generation of HER2 specific mouse CAR T cells: (**A**) Schematic diagram illustrating the modular composition of the retroviral vector encoding HER2 specific CAR. (**B**,**C**) Representative flow cytometry dot-plots and summary data (HER2 CAR mouse T cells (*n* = 8) and non-transduced (NT) mouse T cells (*n* = 8)).

The CAR contains an scFv obtained from trastuzumab, an IgG1 CH2-CH3 extracellular stalk, a CD28 costimulatory endodomain and a CD3z effector domain (Figure 2A). Using trastuzumab as a recognition domain allowed us to compare the impact of CAR T cells as living drugs with the impact of antibodies. The mean transduction efficiency was 8.7% (range: 5.58–11.84%; *n* = 8) as judged by flow cytometric analysis of the HER2 specific scFv (Figure 2B,C). We confirmed that CARs are stably expressed and re-confirmed CAR expression on day 10.

### 2.2. HER2 Specific CARs Redirect Mouse T Cells to HER2 Positive Trastuzumab Resistant Tumor Cells

**Figure 3:**
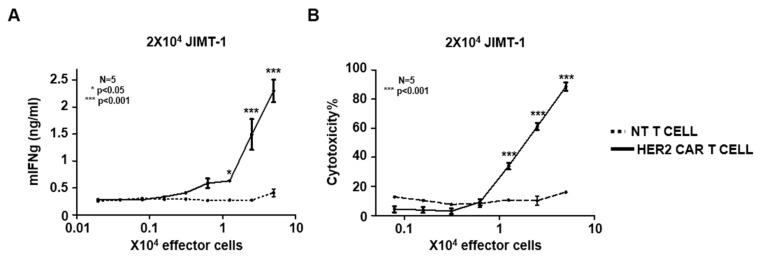
In vitro anti-tumor function of HER2 specific mouse CAR T cells: (**A**) HER2 CAR or non-transduced (NT) mouse T cells were co-cultured with HER2+ JIMT-1 cells at various (2.5:1–0.01:1) T cell to tumor cell ratios. After 48 h, IFNγ release was determined by ELISA (*n* = 2, assay performed in duplicates; HER2 CAR versus non-transduced (NT) T cells: * *p* < 0.05, *** *p* < 0.001). (**B**) XTT-based cytotoxicity assay using HER2 CAR T cells or non-modified mouse T cells and HER2 positive JIMT-1 cells as target at various (2.5:1–0.04:1) T cell to tumor cell ratios (*n* = 2; assay was performed in duplicates; HER2 CAR versus NT T cells: *** *p* < 0.001).

To demonstrate that the HER2 specific CAR redirects mouse T cells to HER2 positive target cells, we co-cultured HER2 CAR T cells with JIMT-1 cells in various effector to target ratios (from 2.5:1 to 0.01:1). HER2 specific CAR T cells recognized the HER2 positive tumor cells indicated by a significant increase in IFNg secretion (*p* < 0.001). Unmodified (NT) T cells did not induce mIFNg release (Figure 3A). While HER2 specific CAR induced T cells killed JIMT-1 tumor cells, no killing was observed in co-cultures with unmodified T cells (*p* < 0.001; Figure 3B). Taken together, the HER2 specific CAR activates mouse T cells in an antigen-dependent manner and induces antigen-dependent tumor cell killing.

### 2.3. HER2 Specific CAR T Cells Have Antitumor Activity In Vivo against HER2+ Trastuzumab-Resistant Tumor Xenografts

**Figure 4:**
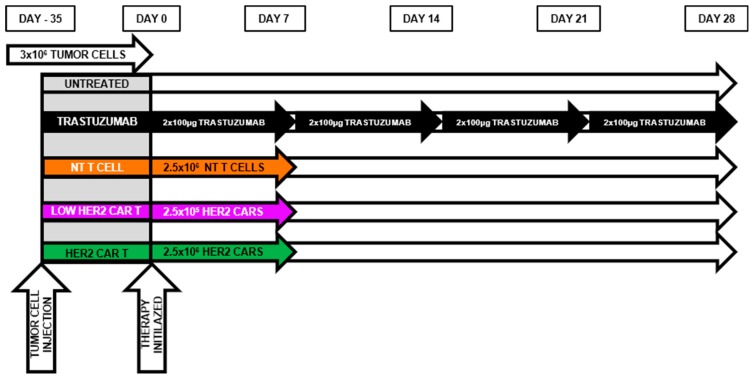
Outline of in vivo animal treatment schedule: Mice were s.c. injected with 3 × 10^6^ JIMT-1 cells. 35 days later (on day 0), mice received 100 µL PBS buffer twice weekly (untreated, *n* = 5), 100 µg trastuzumab in 100 µL PBS buffer twice weekly (trastuzumab, *n* = 5), or an i.v. dose of 2.5 × 10^5^ HER2 CAR mouse T cells (low-dose HER2 CAR, *n* = 5), or an i.v. dose of 2.5 × 10^6^ HER2 CAR mouse T cells (HER2 CAR, *n* = 5). Tumor growth was followed by caliper and was derived as the product of the length, width and height.

**Figure 5:**
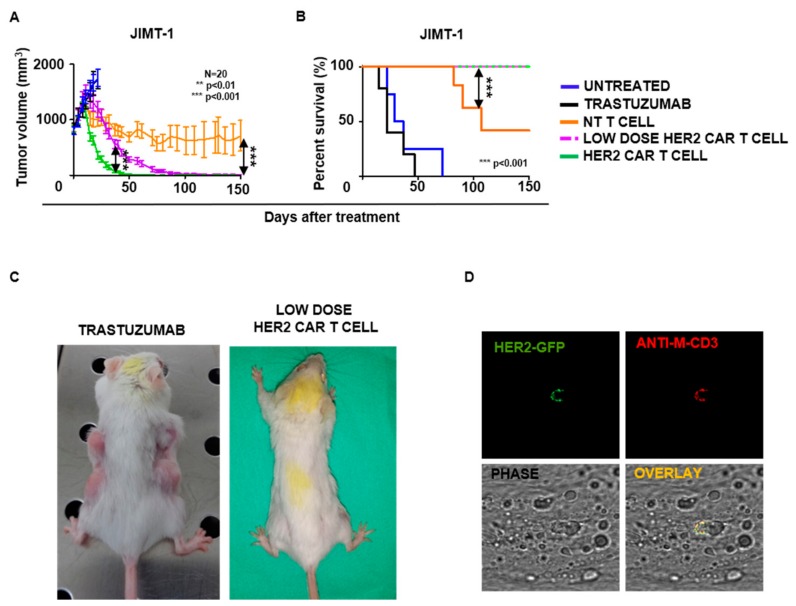
Antitumor activity of HER2-CAR mouse T cells in a xenograft model: (**A**) Quantitative measurement of tumor volumes (volume = mm^3^; low dose HER2-CAR versus HER2-CAR groups: *** *p* < 0.001; NT T cell versus HER2-CAR groups *** *p* < 0.001). (**B**) Kaplan-Meier survival curve (NT T cell versus both HER2 CAR groups *** *p* < 0.001). (**C**) Representative images of animals. (**D**) Representative images for the detection of HER2-CAR mouse T cells in JIMT-1 xenografts (field of view: 92 μm × 92 μm). HER2-CAR mouse T cells were visualized by AlexaFluor647 conjugated anti-mouse CD3e and HER2-GFP co-staining.

To compare the anti-tumor function of antibody treatment and HER2-redirected CAR T cells, we established subcutaneous JIMT-1 xenografts (3 × 10^6^ cells) in SCID mice (day −35, Figure 4). Mice were injected with 100 µg trastuzumab intraperitoneally twice weekly from day 0 (35 days after tumor cell injection), when average tumor size reached 800 mm^3^. Control animals were injected with PBS (Figure 4) and did not show delayed tumor growth and consequently their overall survival was not improved (*p* = 0.79, Figure 5). These data are in line with our previous observations [13]. 

To evaluate the in vivo efficacy of HER2-CAR T cells against these trastuzumab resistant xenografts, 35 days after JIMT-1 inoculation mice were injected iv. with a single dose of 2.5 × 10^6^ (HER2-CAR T cell group), or 2.5 × 10^5^ (Low Dose HER2-CAR T cell group) congenic mouse T cells, 7% of them expressing the HER2-CAR. Control mice were treated with 2.5 × 10^6^ unmodified T cells (NT T cell group) (Figure 4). In this group, the high number of mouse T cells, in some cases, delayed the progression of the human xenografts resulting in better overall survival in comparison to the untreated group (Figure 5A–C). In contrast, the same dose of mouse T cells, when transduced with the CAR at 7% efficiency, completely eradicated the tumors in 57 days and resulted in long-term tumor-free survival. Moreover, complete tumor regression was also observed, by day 105, in the low dose HER2-CAR T cell group in which animals received only 250,000 T cells, among them 17,500 CAR T cells (Figure 5A–C). There is a cell dose dependence of the rate of tumor regression (*p* < 0.001). Despite the difference in time to complete regression, all CAR T cell treated mice remained tumor free until the termination of the experiment (day 150). To assess on-target off-tumor toxicity, formaldehyde-fixed paraffin-embedded tissue section weremade from the heart and lungs of each sacrificed animal. HE-stained sections were characterized based on morphology by an expert histopathologist and showed no signs of mononuclear infiltration (Figure S1A,B). Visual inspection upon dissection also did not show signs of inflammation.

To demonstrate that HER2 specific CAR T cells penetrated the JIMT-1 xenografts, tumor samples from week 3 after CAR T cell injection were immunostained and analyzed by confocal microscopy. We found T cells positive for the CAR and mouse-CD3e confirming the presence of CAR T cells in tumor xenografts (Figure 5D). Taken together, we conclude that HER2 specific CAR T cells have potent in vivo antitumor activity and penetrate HER2 positive xenografts, which are not eliminated by trastuzumab treatment. 

## 3. Discussion

In this study, we described the generation of HER2 specific CAR-modified mouse T cells that obtained their antigen specificity from trastuzumab, a HER2 specific monoclonal antibody applied in clinical practice. We demonstrated that these cells specifically recognize trastuzumab resistant HER2 positive JIMT-1 target cells in vitro. Moreover, in vivo a low dose of HER2 CAR T cell expressed potent anti-tumor activity in a trastuzumab-resistant mouse xenograft model.

Although HER2 specific CAR T cells are effective against breast cancer cells [19,22], the tumor cells preferentially used as targets were trastuzumab sensitive cell lines (SKBR3 or BT474) leaving the question unanswered whether trastuzumab-resistant xenografts can be successfully treated with trastuzumab-derived CAR T cells. 

Incorporation of the trastuzumab scFv into the CAR backbone allowed us to compare the efficacy in tumor eradication by cytolytic CAR T cells versus antibody mediated cytotoxicity.

SCID mice exhibit natural killer cell (NK) activity [23], through which trastuzumab treatment induces antibody dependent cellular cytotocixity against therapy sensitive xenografts (MCF7; BT-474) [24], which makes a direct comparison possible. In vivo, we confirmed that trastuzumab treatment has no potential to delay or revert the growth of established JIMT-1 tumors. which is in line with previous observations [11,12,13,14].

Although unmodified mouse T cells in co-cultures with JIMT-1 cells did not release cytokine and did not exhibit cytotoxicity, we wanted to confirm that xenogenic immune response does not reject human tumor xenografts in vivo [23]. A single injection of 2.5 × 10^6^ unmodified mouse T cells on day 35 following JIMT-1 implantation could not eradicate the human tumor xenografts; however, in some cases, it caused tumor regression and resulted in stable disease. 

In contrast to the control and trastuzumab-treated groups, 2.5 million mouse T cells with a proportion of 7% CAR-transduced cells (total ~175,000) caused a complete remission in 57. Even a tenth of this dose, 250,000 T cells including ~17,500 CAR T cells, was fully curative, although only in 105 days. The number of CAR T cells yielding complete remission in the latter case is only 0.2–0.3% of the usual 5 to 10 million CAR T cells used in successful mouse CAR-T therapy models [25]. Thus, it is likely that this small fraction of specifically redirected T lymphocytes successfully penetrates the tumor mass and evokes, in addition to direct tumor killing, a focusing effect that concentrates the xenogenic response of non-transduced mouse lymphocytes against the human tumor. By extrapolation, it is possible that in the case of HER2 positive solid tumors, a reduced number of CAR T cells could still maintain therapeutic efficacy through actively penetrating the tumor and enhancing the activity of tumor infiltrating lymphocytes (TILS, [26]). At the same time, while "on target off tumor” toxicity (mainly in the cardiopulmonary system), could be avoided owing to the lower expression of the HER2 target and the lack of TILs in healthy tissues.

Overall, we conclude that even a small number of CAR T lymphocytes can evoke a robust anti-tumor response against an antibody resistant xenograft by focusing the activity of xenogenic T cells. This observation may have significance for optimizing the dose of CAR T cells in the therapy of solid tumors. 

## 4. Materials and Methods 

All materials were from Sigma-Aldrich (St. Louis, MO, USA) unless otherwise indicated.

### 4.1. Cells and Culture Conditions

HEK 293T packaging cells were purchased from the American Type Culture Collection (ATCC, Manassas, VA, USA). Cells were cultured in Dulbecco’s Modified Eagle Medium (DMEM) supplemented with 2 mmol/L Glutamax and 10% Fetal Calf Serum (FCS) and antibiotics. The JIMT-1 human breast cancer cell line was established in the laboratory of Cancer Biology, University of Tampere, Finland [12]. These cells were cultured in 1:1 ratio of Ham’s F-12 and DMEM supplemented with 20% FCS, 300 U/L insulin, 2 mmol/L GlutaMAX and antibiotics. Primary mouse splenocytes, T cells and CAR T cells were cultured in RPMI 1640 supplemented with 2 mmol/L GlutaMAX, 10% FCS and antibiotics. All of the above-listed cells and cell lines were maintained in a humidified atmosphere containing 5% CO_2_ at 37 °C and were routinely checked for the absence of mycoplasma contamination.

### 4.2. Retrovirus Production and Transduction of T Cells

Retroviral particles were generated by transient transfection of HEK 293T cells with the MSGV retroviral vector containing a trastuzumab derived HER2 specific CAR [17], a Peg-Pam-e plasmid containing the sequence for MoMLV gag-pol, and a pMEVSVg plasmid containing the sequence for VSVg, using jetPrime transfection reagent (Polyplus, Illkirch, France). Supernatants containing the retrovirus were collected after 48 h (Figure 1A). 

To generate HER2 specific CAR T cells, T cells of syngenic Balb/c mice were isolated from a freshly dissected spleen by using a mouse-specific T cell isolation MACS sorting kit (130-095-130; Miltenyi Biotech; Bergisch Gladbach, Germany). MACS sorted mouse T cells were plated on non-tissue culture treated 24-well plates (5 × 10^6^ cells/well), which were pre-coated with 1µg/manti-mouse CD3e (ThermoFischer, Waltham, MA, USA) and anti-mouse CD28 (R&D Systems, Min L neapolis, MN, USA) antibodies. After 24 h, mouse interleukin 2 (mIL2; 700 U/ml) was added to cultures. T cells were then transduced with the previously described retroviral particles on RetroNectin-coated (Takara, Kusatsu, Japan) plates on day 3 in the presence of mIL2 (200 U/m L). The expansion of T cells was subsequently supported with mIL2. Anti-mouse CD3e/CD28 activated non-transduced (NT) T cells were expanded in parallel with mIL2. Following 48-72h incubation, cells were collected and used for further experiments (Figure 1B).

### 4.3. Flow Cytometry

HER2 specific CAR expression was confirmed by a HER2-GFP recombinant protein. T cell purity was determined by Alexa Fluor 647 conjugated anti-mouse CD3 antibody (BD Biosciences, San Jose, CA, USA) staining. Both molecules were used at 10 µg/mL final concentration for 10 minutes on ice. Analysis was performed on at least 10,000 cells per sample using a FACS Calibur (Becton Dickinson, Franklin Lakes, NJ, USA) instrument and FCS Express 6 software (De Novo Software, Glendale, CA, USA).

### 4.4. CAR-Mediated T Cell Activation

Mouse CAR T cells and non-transduced controls were cultivated in indicator-free RPMI 1640 medium and 10% (*v*/*v*) FCS, without additional stimuli for 24  h, washed and incubated on 96-well round-bottomed plates in the presence of JIMT-1 target cells for 48  h. Culture supernatants were analyzed for IFN-γ by ELISA (BD Biosciences). IFN-γ was bound to a solid-phase mAb R46A2 and detected by a biotinylated mAb XMG1.2. The reaction product was visualized by a peroxidase-streptavidin conjugate (1:10,000) and ABTS as substrate. To monitor the cytolytic activity, genetically modified and control T cells were co-cultured with JIMT-1 target cells with increasing T cell numbers for 48 h in 96-well round-bottomed plates. Specific cytotoxicity was monitored by a 2,3-bis[2-methoxy-4-nitro-5-sulphophenyl]-2H-tetrazolium-5-carboxanilide salt (XTT)-based colorimetric assay (‘Cell Proliferation Kit II’, Roche Diagnostics, Risch, Switzerland). Reduction of XTT was determined as OD at 480 nm for treated tumor cells (Tu), for untreated tumor cells (Max) and for T cells only (T). Background (Bg) was measured in complete medium with XTT but no cells. Measurements were run with minimally 3 technical replicates in three independent experiments. Cytotoxicity was calculated as (1).

(1) $$\mathrm{Cytotoxicity}\;\left(\%\right)=\left(1-\frac{Tu-T}{Max-Bg}\right)\cdot 100\%.\;$$

### 4.5. Xenograft Tumors and In Vivo Antibody Treatment

SCID (C.B-17/Icr-Prkdc^scid^/IcrIcoCrl, Fox-Chase) mice were purchased from Charles River Laboratories, and housed in a specific-pathogen-free environment. All animal experiments were performed in accordance with FELASA guidelines and recommendations and DIN EN ISO 9001 standards. Only non-leaky SCID mice with murine IgG levels below 100 ng/mL were used. Each seven-week-old female SCID mouse participating in the study was given a subcutaneous injection in both flanks, each containing 3 × 10^6^ JIMT-1 cells suspended in 100 µL PBS buffer and mixed with an equal volume of Matrigel (BD Biosciences, San Jose, CA, USA). Tumor volumes were derived as the product of the length, width and height measured with a caliper. 

The trastuzumab group was treated with 100 µg trastuzumab intraperitoneally in 100 µL PBS twice weekly from day 35 post tumor cell injection. The untreated control group was injected with 100 µL PBS buffer i.p. twice weekly. In the HER2 CAR T cell and unmodified mouse T cell groups mice received a single dose of 2.5 × 10^6^ effector cells i.v. on day 35 post JIMT-1 inoculation. In the low dose HER2 CAR T cell group mice received 2.5 × 10^5^ cells i.v. (Figure 3A). At the end of the experiment, the animals were euthanized. Experiments were approved by the National Ethical Committee for Animal Research (# 5-1/2018/DEMÁB). 

### 4.6. Tumor Xenograft Sections

At termination, mice were dissected, and fresh tumors were embedded in cryomatrix (Thermo Fischer Scientific, Waltham, MA, USA) and snap-frozen in isopentane submerged in liquid nitrogen. Serial 14 µm thick cryosections were made with a Shandon Cryotome (Thermo Fischer Scientific, Waltham, MA, USA) at −24 °C and air-dried. Staining was carried out at room temperature and all labeling molecules were diluted in PBS buffer supplemented with 1% BSA. After 5 min of rehydration in PBS buffer containing 1% BSA and 0.01% TritonX-100 (Thermo Fischer Scientific, Waltham, MA, USA) HER2 CAR mouse T cells were stained with HER2-GFP recombinant protein and Alexa Fluor 647 conjugated anti-mouse CD3e antibodies. Both molecules were used at 2 µg/mL concentration at 4 °C for 10 h. Sections were washed three times, for 5, 20, and 60 minutes, fixed in formaldehyde, and mounted in Mowiol antifade.

### 4.7. Haematoxylin and Eosin Stained Sections

The heart and lung were resected from sacrificed mice, fixed in 4% paraformaldehyde for 4–6 h, dehydrated in ethyl alcohol, and embedded in paraffin. Serial sections of 6 μm were cut with a microtome. Deparaffinized sections were HE stained using standard procedures and imaged using a Pannoramic digital histopathology scanner with a 20× objective in transmission mode. A trained histopathologist has examined the digital slides.

### 4.8. Confocal Laser Scanning Microscopy

Immunofluorescence-labeled tissue sections were analyzed with a confocal laser scanning microscope (LSM 510, Carl Zeiss GmbH, Jena, Germany) using a 40× C-Apochromat water immersion objective (NA = 1.2). GFP was excited at 488 nm and Alexa Fluor 647 at 633 nm. Corresponding fluorescence emissions were separated with an appropriate quad-band dichroic mirror, and detected through 505 to 550 nm bandpass and 650 nm longpass filters, respectively. Pinhole was set for 4 µm thick optical sections.

### 4.9. Statistical Analysis

GraphPad Prism 5 software (GraphPad software, Inc., La Jolla, CA) was used for statistical analysis. Data were presented as mean ± SD or ± SEM. For comparison between two groups, a two-tailed *t*-test was used. For comparisons of three or more groups, one-way ANOVA with Bonferroni’s post-test was used. For the mouse experiments, survival, determined from the time of tumor cell injection, was analyzed by the Kaplan-Meier method and log-rank test. *p*-values < 0.05 were considered statistically significant.

## Supplementary Materials

Supplementary materials can be found at https://www.mdpi.com/1422-0067/21/3/1039/s1.

## Abbreviations

CAR	Chimeric antigen receptor
GFP	Green fluorescent protein
HER2	Human epidermal growth factor receptor 2
IFN-γ	Interferon gamma
PBS	Phosphate buffered saline
scFv	Single Chain Variable Fragment
SCID	Severe combined immunodeficiency
