# Supplementary material for: A Small Number of HER2 Redirected CAR T Cells Significantly Improves Immune Response of Adoptively Transferred Mouse Lymphocytes against Human Breast Cancer Xenografts
Source: Int J Mol Sci. 2020 Feb 4;21(3):1039. doi: 10.3390/ijms21031039 (PMC7038081; doi:10.3390/ijms21031039)
Supplement: Supplementary file 1 [file ijms-21-01039-s001.pdf]

Supplementary figure

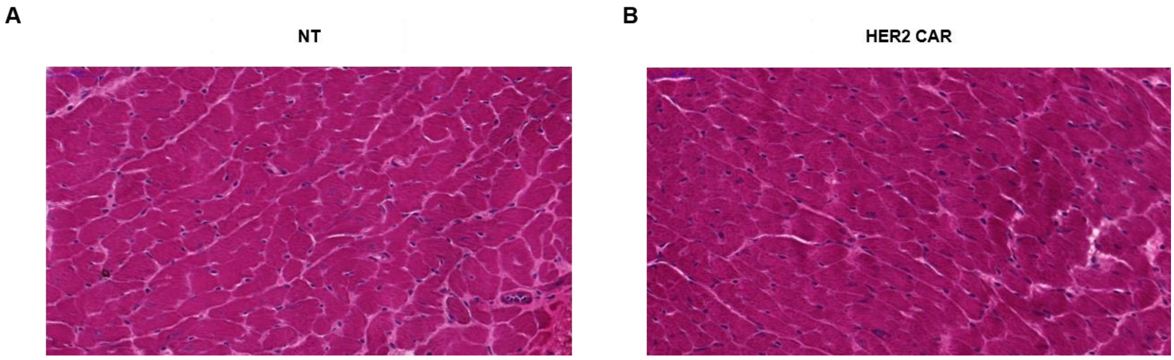

Supplementary Figure 1. Analysis of systemic toxicity by histology: Heart tissues harvested from NT T cell treated mice (**A**) and mice treated with HER2 CAR T cells (**B**) were examined after HE staining.
